# DNA Methylation in the Fields of Prenatal Diagnosis and Early Detection of Cancers

**DOI:** 10.3390/ijms241411715

**Published:** 2023-07-20

**Authors:** Fabio Coppedè, Utsa Bhaduri, Andrea Stoccoro, Vanessa Nicolì, Eleonora Di Venere, Giuseppe Merla

**Affiliations:** 1 Department of Translational Research and of New Surgical and Medical Technologies, University of Pisa, 56126 Pisa, Italy; 2 Interdepartmental Research Center of Biology and Pathology of Aging, University of Pisa, 56126 Pisa, Italy; 3 Laboratory of Regulatory & Functional Genomics, Fondazione IRCCS Casa Sollievo della Sofferenza, San Giovanni Rotondo, 71013 Foggia, Italy; 4 Department of Life Sciences, University of Trieste, 34127 Trieste, Italy; 5 Department of Molecular Medicine & Medical Biotechnology, University of Naples Federico II, 80131 Naples, Italy

**Keywords:** DNA methylation, DMNT, bisulfite sequencing, RRBS, epigenetics, cancer

## Abstract

The central objective of the metamorphosis of discovery science into biomedical applications is to serve the purpose of patients and curtail the global disease burden. The journey from the discovery of DNA methylation (DNAm) as a biological process to its emergence as a diagnostic tool is one of the finest examples of such metamorphosis and has taken nearly a century. Particularly in the last decade, the application of DNA methylation studies in the clinic has been standardized more than ever before, with great potential to diagnose a multitude of diseases that are associated with a burgeoning number of genes with this epigenetic alteration. Fetal DNAm detection is becoming useful for noninvasive prenatal testing, whereas, in very preterm infants, DNAm is also shown to be a potential biological indicator of prenatal risk factors. In the context of cancer, liquid biopsy-based DNA-methylation profiling is offering valuable epigenetic biomarkers for noninvasive early-stage diagnosis. In this review, we focus on the applications of DNA methylation in prenatal diagnosis for delivering timely therapy before or after birth and in detecting early-stage cancers for better clinical outcomes. Furthermore, we also provide an up-to-date commercial landscape of DNAm biomarkers for cancer detection and screening of cancers of unknown origin.

## 1. DNA Methylation: Looking Back over the Century

*“Genes are equivalent to blueprints; epigenetics is the contractor. They change the assembly, the structure.”*

—Bruce Lipton.

The methylation of the DNA is a quintessential epigenetic mechanism consisting of the binding of a methyl group to the fifth carbon of the cytosine residue, often located in a CpG dinucleotide, leading to the formation of a 5-methylcytosine, or, in short, 5-mC. The study of DNA methylation has played a significant role in molecular biology, particularly in broadening the horizons of classical genetics into modern epigenetics. From a historical perspective, the discovery of 5-methylcytosine in living cells was originally carried out by Johnson and Coghill in 1925 [1]. In their attempt to understand the *Mycobacterium tuberculosis* pathogenic mechanism, they reported the presence of 5-mC for the first time in their microscopic analysis of pyrimidine picrate crystals when they isolated and crystallized nucleic acids from bacteria [2].

Despite this astounding discovery, it took 23 gap years for a second study on 5-mC to appear in the literature, and it was in 1948 when Hotchkiss found 5-mC in hydrolyzed calf thymus DNA by paper chromatography [3]. In his quantitative separation study of purines, pyrimidines, and nucleosides by means of paper chromatography, Hotchkiss noticed a modified cytosine that pretended to be cytosine but had slightly more migration, and in terms of its mobility and ultraviolet absorption spectrum, it behaved exactly in the manner that thymine does to uracil. This convinced Hotchkiss to deduce the modified cytosine as the plausible 5-methylcytosine because thymine is nothing but 5-methyluracil, and hence he labeled it as “epi-cytosine” [3]. Consequentially, this occurrence of 5-methylcytosine in nucleic acids was further verified by Wyatt in 1950 using paper chromatography in plant and mammalian sources [4,5].

Now, this is noteworthy because this juncture of the last century had already started observing a philosophical shift to understand gene regulation outside the frame of classical genetics. The embryologist Conrad Waddington was the first to mention the term ‘epigenetics’ in order to describe “the complex of developmental processes between the genotype and phenotype” and throw light on how genes can possibly interact with the environment to shape the phenotype of an organism [6]. Simultaneously, many studies came out with propositions that DNA methylation could be involved in the regulation of gene expression, but it was only during the 1980s that many reports started showing that DNA methylation can actually be involved in gene regulation [7,8,9].

Nowadays, nearly a century later since its discovery in 1925, it is well established that DNA methylation is an essential epigenetic control mechanism in mammals alongside the other epigenetic regulators [10] and that it is involved in several developmental processes like embryogenesis [11], genomic imprinting [12], and multiple human diseases like cancer [13], neurodevelopmental, neurodegenerative, and neuropsychiatric disorders [14]. Following the progress in human genomics, and the advances in methylation sequencing, the 5-mC has begun to be called the “fifth base” of the genome [15]. However, the scope of DNA methylation in epigenetics is no longer limited to 5-mC since the discovery of two other DNA modifications in humans with gene expression regulatory functions, namely 5-hydroxymethylcytosine (5-hmC) and N6-methyladenine (6-mA) [15].

In mammals, 5-mC DNA methylation is frequently found in CpG islands, which are DNA sequences particularly enriched in CpG dinucleotides and are mainly located in the promoter and exonic regions of roughly 40% of mammalian genes. Methylation of CpG dinucleotides is performed by three DNA methyltransferases (DNMTs), DNMT1, DNMT3A, and DNMT3B, which use S-adenosyl-L-methionine (SAM) as the source of methyl groups [16]. A normal level of DNA methylation is a crucial requirement to regulate the expression of the paternal and maternal alleles of genes under the control of imprinting during development [12], whereas aberrant DNA methylation is a widespread signature in cancers that affects the regulation and expression of tumor suppressor genes [13].

In this review, the growing sphere of DNA methylation techniques and the applications of DNA methylation studies in the prenatal diagnosis of human diseases like imprinting disorders and chromosomal abnormalities have been discussed. In addition, the implications of DNA methylation as a diagnostic tool in early cancer care are also reviewed from an epigenetic point of view, with an emphasis on sporadic and hereditary tumors.

## 2. DNA Methylation Techniques

### 2.1. Methods Based on Methylation-Sensitive or -Dependent Restriction Enzymes (MSRE/MSDE)

**Figure 1:**
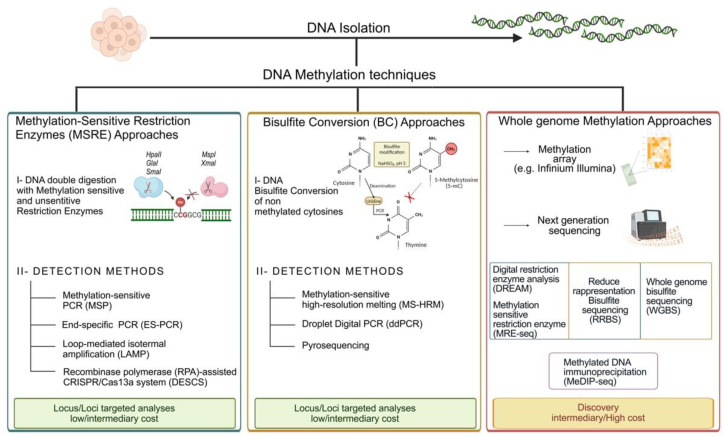
Summary of the major techniques used to analyze DNA methylation. The Blu panel includes methods based on DNA digestion with methylation-sensitive and -insensitive restriction enzymes. The yellow panel shows the detection methods for DNA methylation based on sodium bisulfite pre-treatment to convert unmethylated cytosines into uracils and then, after the PCR cycles, into thymines. In the red panel, the whole genome methylation approaches are indicated. They can be divided into array and sequencing-based (new generation sequencing, NGS) methods. NGS technologies are based on different pre-treatments of the DNA samples. The panel reports some of the most used methodologies, including sensitive restriction enzymes (MRE-seq) and digital restriction enzyme analysis (DREAM) (blusquare), affinity enrichment approaches based on immunoprecipitation with a methylcytosine-specific antibody (MeDIP-seq) (purple square) and bisulfite conversion-based methods (yellow square). Among the three, the most adopted is DNA sequencing following bisulfite conversion.

The development of techniques for the characterization of the DNA methylation profile started around 1980 with non-specific methods for methylation detection through reversed-phase high-performance liquid chromatography (RP-HPLC) [17]. This technique and its subsequent modifications allowed to establish the ratio of methylated cytosines versus unmethylated ones. Moreover, several technologies were applied in these decades to increase the resolution and attempt to discriminate the methylation within a specific region with a single base specificity [18]. Over these decades, three major chemical biology approaches, the “methylation-sensitive restriction enzyme (MSRE) approach”, “bisulfite conversion (BC) approach” and “whole genome methylation approach” have been used for the detection of DNA methylation (Figure 1). These major approaches can further be divided among different techniques depending on the usage of microarray or sequencing-based methods for addressing the biological query [19,20]. An early approach, still the basis of some valid techniques currently adopted, involves the use of enzymes that are sensitive to the methylation marks on the DNA sequence. The first isoschizomer pairs adopted were *HpaII* and *MspI*, which recognize and cut the same sequence: CCGG [21]. In fact, the methylation of the second C of the target motif enables the cut promoted by *MspI* and inhibits the activity of *HpaII*. Initially, the visualization of the digested DNA was made through radiolabeling and chromatography-based techniques. Nowadays, the detection of digested fragments is frequently achieved by methylation-sensitive PCR (MSP) and electrophoretic analyses of the product size. Thus, the approaches of MSRE-based techniques rely on enrichment of methylated fragments following enzymatic degradation of unmethylated fragments. Concerning the detection of the methylation percentage of the studied region, it can be made, for example, using quantitative PCR (qPCR) [22], loop-mediated isothermal amplification (LAMP) [23], or recombinase polymerase amplification (RPA)-assisted CRISPR/Cas13a system (DESCS) [24].

Another example is the Digital Restrictive Enzyme Analysis of Methylation (DREAM) [25], a procedure that allows the quantitative recording of DNA methylation across the genome using next-generation sequencing (NGS) technology. The procedure entails the use of *SmaI* and *Xmal* enzymes, the first ones which are specific for the unmethylated form of the target sites CCCGGG that are initially digested, forming 5′-GGG. Thus, the enriched methylated sites are cut by *Xmal*, forming 5′-CCGG overhangs. The sequencing libraries prepared allow to determine the methylation at a single CpG site as the ratio of the sequencing reads with the methylated tags to the total reads mapping on the sites [25].

On the other hand, an example of the evolution of the MSDE methods is end-specific PCR (ES-PCR), in which the amplification of the DNA occurs exclusively among the sample cut by the methylation-sensitive enzyme *GlaI*. A very recent work proposed an update in the detection of the methylation percentage of the samples, proposing a further extension of terminal transferase (TdT) at the 3′-end of the methylated-cut fragments, serving as template for the following qPCR [26]. This method of detection was tested on colorectal cancer tissue samples, resulting in a more advantageous results than the previous methods adopted [27,28], which are limited by low amplification efficiency or false positive signals. Despite the widespread use of restriction enzymes in diagnostic practice, the lack of robustness, the amount and the quality of genomic materials required, and their semiquantitative nature constitute the limitations of these enzyme-based methods.

### 2.2. Bisulfite-Based Methods

Nowadays, the gold standard techniques used to evaluate DNA methylation patterns are based on sodium bisulfite (SB) conversion, the fundamental preliminary step of all these methods, as proposed by Frommer in 1992 [29]. The SB treatment is a very simple and relatively fast process that mediates the conversion of the cytosine into uracil through its deamination. At the end of the procedure, all methylated cytosines will be protected by the treatment and read as cytosines, while unmethylated cytosines will be translated into thymine after a PCR step. Thus, BS treatment reduced the complexity of the genome, although it led to fragmentation of the treated DNA making the amplification of the longest fragments potentially difficult. However, outside the context of the sequencing, the technologies adopting the BS treatment were the methylation-specific polymerase chain reaction (MSP), based on primers able to discriminate between methylated and unmethylated regions, and the methylation-sensitive high-resolution melting (MS-HRM), in which distinct melting profiles are generated between methylated and unmethylated amplicons.

Droplet Digital PCR (ddPCR) is a recent advance in DNA methylation studies characterized by higher sensitivity and precision than conventional PCR-based methods. For instance, the generation of standard curves is not required because the count of target molecules occurs directly [30]. It is one of the methods used for the detection of circulating levels of DNA in the plasma of cancer patients [31]. Finally, pyrosequencing is a sequencing-by-synthesis method currently considered the gold standard for methylation pattern studies. This method allows quantitative measurements in real time of the nucleotide incorporation by detecting the light signals generated by the release of pyrophosphates. The degree of methylation for each CpG position analyzed is defined as the T/C ratio. This technique is characterized by great reproducibility and accuracy and high quantitative resolution [32] and has therefore been proposed as a method for promoter methylation analysis in routine clinical practice for onco-targets, such as *MGMT* [33].

### 2.3. Whole Genome Methylation Approaches

DNA methylation arrays and sequencing approaches are the two key technologies used for the analysis of genome-wide DNA methylation. Methylation arrays, such as the Illumina Infinium ones, are very sensitive, specific, and reproducible methods to analyze the methylation levels of >930,000 CpG sites with a competitive cost respect to the sequencing methodologies, and these factors make their application optimal for the analysis of cancer methylomes. On the other hand, sequencing-based technologies allow us to explore methylation patterns with far greater resolution than DNA methylation arrays. These methods could be based on different approaches, including the use of methylation-sensitive restriction enzymes (MRE-seq), affinity enrichment approaches based on immunoprecipitation with a methylcytosine-specific antibody (MeDIP-seq) [34], and bisulfite conversion-based methods [35]. Among the three, the most adopted is DNA sequencing following bisulfite conversion.

Indeed, whole-genome bisulfite sequencing (WGBS, BS-seq) and reduced-representation bisulfite sequencing (RRBS) approaches allow the analysis of genome-wide methylation patterns after BS treatment of the sample DNA. In WGBS, the whole bisulfite-converted genome is sequenced and elaborated by various bioinformatics protocols [20,36,37]. By contrast, RRBS uses methylation-sensitive enzymatic digestion to capture the methylated regions of the genome, followed by BS processing of the DNA fragments before amplification and sequencing [38]. In both cases, the sequencing could be performed using any existing NGS platform, such as the Illumina and Life Technologies ones. Albeit these procedures can measure alterations in DNA methylation on a genome-wide scale, several limitations should be considered, including the costs and difficulties in the analysis of NGS data as well as the significant amount of DNA needed for the analyses. These items could be partially resolved by choosing RRBS, which interrogates only the CpG-rich portions of the genome that are covered by the enzyme, making it convenient in terms of time, costs, and the low sample input required [39]. RRBS and WGBS are not able to distinguish between 5 mc and 5 hmc in the analysis. However, oxidative bisulfite sequencing (oxBs-seq) approaches, which consist of the specific chemical oxidation of 5 hmC to 5-formylcytosine (5 fC) prior to bisulfite treatment, allow discriminating between 5 mC and 5 hmC at a single-base resolution in genomic DNA [40]. Furthermore, single-cell RRBS (scRRBS) and single-cell WGBS (scWGBS) approaches have been developed in order to overcome the limitations of cellular heterogeneity in tissues and tumors [41]. Overall, RRBS and WGBS constitute the best options for the detection of novel diagnostic and prognostic biomarkers, for the prediction of response to therapy, and for many other applications in translational medicine [42]. Moreover, these approaches lead to the generation of several datasets, including genetic information about many cancer types and neurodevelopmental disorders, which are available for further analysis [20].

## 3. DNA Methylation in Prenatal Diagnosis

### 3.1. DNA Methylation as a Biomarker for Fetal DNA Enrichment and Non-Invasive Prenatal Testing (NIPT)

Non-Invasive Prenatal Testing (NIPT) is a clinical procedure for the detection of certain chromosomal abnormalities based on the analysis of cell-free fetal DNA (cffDNA) in maternal blood during early pregnancy [43,44], and the procedure is not harmful to the fetus. The cffDNA in maternal plasma was detected for the first time in 1997 by Lo et al. [45], and it consists of short DNA fragments [46,47,48,49] that originate from the placenta but represent the entire fetal genotype. In the NIPT approach, the key concept is the ability to separate maternal and fetal DNA molecules because of the shortness of the latter compared to the maternal ones [44]. In recent years, NIPT has been gradually accepted as the screening test not only for its sensitivity (99%) and specificity (99.5%) for analysis of cffDNA to determine the risk of aneuploidies, including trisomy 13 (Patau Syndrome), trisomy 18 (Edwards Syndrome) and trisomy 21 (Down Syndrome), but also for determining fetus RhD status in RhD-negative mothers, fetus sex and single-gene disorders [50,51]. DNA methylation was introduced in the context of NIPT in 2002 when the investigation of methylation differences between fetal and maternal DNA allowed to specifically target fetal DNA in maternal plasma [52].

Over the years, DNA methylation has gained relevance in non-invasive prenatal testing due to the identification of many markers exhibiting differential DNA methylation between the maternal blood cells and the placenta [44]. Among all, it was the hypomethylated SERPINB5 gene (chromosome 18) promoter that became the first universal marker for fetal DNA in maternal plasma [53], enabling for the first time the non-invasive detection of fetal trisomy 18. Afterward, the identification of specific methylation patterns for maternal and fetal DNA in CpG islands on chromosome 21 provided a source of markers for the non-invasive diagnosis of trisomy 21 or Down syndrome [54]. Since then, several fetal epigenetic molecular markers were identified that were previously unknown, with the advantages of high-throughput approaches that allowed to overcome the limitations of conventional studies based on the use of techniques involving restriction enzymes [53,54,55] or bisulfite-converted DNA [55,56]. One of these high-throughput approaches is methylated DNA immunoprecipitation sequencing (MeDIP-seq), which is based on the use of 5 mC-specific antibodies to capture methylated fragments, followed by their sequencing. This enabled the identification of methylated loci on chromosomes 13, 18, 21, X and Y, potentially providing targets for non-invasive prenatal diagnosis of the common aneuploidies [57,58,59,60]. Later, the combination of MeDIP and in-solution hybridization followed by NGS led to the identification and validation of 331 fetal-specific differentially methylated regions (DMR), supplying an important key for the non-invasive investigation of fetal abnormalities in maternal plasma [61]. In case of these NGS-based NIPT approaches, the fetal DNA component plays a crucial role in bringing out an accurate clinical interpretation, and so far, many bioinformatics approaches have been developed and standardized to estimate the fetal DNA fraction [62]. Peng and Jiang (2017) reviewed the major approaches that have been developed for NGS-based NIPT methods to estimate fetal DNA fraction for optimizing the proper clinical interpretation pipeline, e.g., “Y Chromosome-Based Approach”, “Maternal Plasma DNA Sequencing Data with Parental Genotype-Based Approach”, “High-Depth Sequencing Data of Maternal Plasma DNA-Based Approach”, “Shallow-Depth Maternal Plasma DNA Sequencing Data with Maternal Genotype-Based Approach”, “Fetal Methylation Marker-Based Approach”, “Cell-Free DNA Size-Based Approach”, and “Cell-Free DNA Nucleosome Track-Based Approach” [62]. Although there were multiple tools developed with these different approaches, there were no optimized guidelines for different steps of analysis in the clinical context. To address this issue, an automated pipeline called NiPTUNE was developed in an attempt to integrate all steps of NIPT analysis into an open-source Python package. This includes many modules that can be run altogether or independently and that allow features such as estimation of fetal DNA fraction, accurate prediction of gender and fetal aneuploidies [63]. It is noteworthy that until now, the United States Food and Drug Administration (FDA) has not held any regulatory authority on NIPTs as they are considered as Laboratory-Developed Test (LDT), and FDA has maintained a “general policy of enforcement discretion” for most LDTs since the implementation of the Medical Device Amendments in 1976. It means current NIPTs that are in use are not reviewed by the FDA [64]. Nevertheless, the cffDNA-based NIPTs are recognized as the most efficient prenatal screening option to detect genetic abnormalities and the most common serious chromosomal diseases in the fetus such as trisomy 13, trisomy 18 and trisomy 21 [65]. In 2023, the International Society for Prenatal Diagnosis (ISPD) reported its latest public position statement on NIPTs for fetal chromosomal conditions in singleton pregnancies, replacing their earlier statement published in 2015 [66]. The ISPD consensus statement reads, “NIPT is the most accurate screening test for the common autosomal aneuploidies (trisomies 21, 13 and 18) in unselected singleton populations”. For trisomy 13, trisomy 18, and trisomy 21, the ISPD reported the high performance of NIPT with a sensitivity of 100% and a specificity of 99.96% for trisomy 13, a sensitivity of 98.83% and a specificity of 99.93% for trisomy 13, and a sensitivity of 98.80% and a specificity of 99.96% for trisomy 21 [66].

### 3.2. DNA Methylation in Imprinting Disorders Diagnosis and Assisted Reproductive Technology (ART) Impact

Human-assisted reproductive technology (ART), notably in vitro fertilization (IVF) and intracytoplasmic sperm injection (ICSI), is correlated with an increased incidence of some rare imprinting disorders, including Beckwith-Wiedemann syndrome (BWS), Angelman syndrome (AS), Silver-Russell syndrome (SRS), and Prader-Willi syndrome (PWS) [67,68,69,70,71]. The genomic imprinting and epigenetic reprogramming are two important processes for embryogenesis that occur in the first steps of fertilization and germ line generation and are regulated by two major waves of genome-wide demethylation and remethylation [72]. Several studies showed that ART might expose the developing epigenome to several environmental cues, such as artificial hyperstimulation of ovulation, culture conditions of embryos, embryo cryopreservation and embryo transfer, and all these manipulations overlap with epigenetic reprogramming and sex-specific imprinting acquisition events [72,73]. For example, it has been reported in different studies that the risk of BWS increased by about three- to six-fold in children born through ART [68,70]. Nevertheless, it should be noted that abnormal DNA methylation in ART children could not be identified consistently. Actually, along with this, some reports also gave another explanation for the higher rate of imprinting disorders in children conceived by ART due to some pre-existing imprinting errors in the sperm of infertile men, particularly those with oligozoospermia [73].

Besides these imprinting disorders related to genes regulated according to parent-of-origin, DNA methylation disturbances are associated with multi-locus imprinting disturbance (MLID). Recently, Anvar et al. started to shed light on the pivotal role of epigenetic regulation during the preimplantation phases, which is greatly susceptible to issues related to maternal physiology or ART procedures, potentially predisposing to disorders related to aberrant genomic imprinting [74]. A novel approach, namely ImprintSeq, proposed by Ochoa et al., improved the diagnosis and research investigations of congenital imprinting disorders (CIDs) and MLIDs. This is an accurate and quantitative high-throughput method to interrogate multiple imprinted differentially methylated regions (iDMRs) at the same time by exploiting a methylation sequencing panel that covers the most relevant iDMRs for CID and MLID detection [75].

## 4. DNA Methylation Test in Early Cancer Diagnosis

Cancer is a multistep process during which several pathways operate together for the initiation and progression of the tumor. During the tumorigenesis process, epigenetic changes play a role in early cellular alterations by changing the expression of tumor-associated genes, including hypermethylation of tumor suppressor genes and hypomethylation of oncogenes, as well as global DNA hypomethylation [76]. The pivotal role of DNA methylation in cancer etiology is well documented by some cases of hereditary tumors in which constitutional epimutations predispose to cancer. Such mechanisms have been reported for retinoblastoma, Lynch syndrome, breast cancer and chronic lymphocytic leukemia [77,78,79,80].

Research on epigenetic alterations and particularly DNA methylation in peripheral tissues, including blood, saliva, or urine, is proving to be of great help in the clinical practice of sporadic cases of cancer. Indeed, several advancements have been made in the implementation of clinical epigenetics in the oncology field and several epigenetic biomarkers for cancer diagnosis have been proposed in recent years [81].

Notably, in recent years, epigenetic biomarkers in liquid biopsies, i.e., cell-free DNA in body fluids that can be obtained in a non-invasive manner, such as peripheral blood, urine or saliva, have allowed the detection of biomarkers for a broad range of cancers [82]. Currently, some commercial kits for cancer detection that are designed to evaluate DNA methylation levels at specific loci are already available, and several others are under clinical evaluation for their implementation in the market (Table 1 and Table 2).

The first commercially or clinically available test based on DNA methylation for CRC screening was ColoSure™ [83]. This test was designed to detect CRC by evaluating fecal DNA methylation of the vimentin (*VIM*) gene. A few years later, another test for CRC diagnosis, called ColoGuard, which received approval from the United States Food and Drug Administration (FDA), was based on DNA methylation analysis in stool samples [84]. In addition to the evaluation of *NDRG4* and *BMP3* gene methylation, this kit also includes the screening of *KRAS* mutations and the hemoglobin immunological dosage. For ColoGuard, it has been reported to have a sensitivity and a specificity of 0.92 and 0.87, respectively, for the detection of CRC, so it can detect significantly more cancers compared to fecal immunochemical tests, but also leads to more false positive results (13%). The assay can also detect 42% of high-risk precancerous lesions [84].

Two commercially available kits, the EarlyTect-Colon Cancer and Colosafe, which respectively received approval from the Korean Food and Drug Administration and the China National Medical Products Administration, have been designed to only detect methylated Syndecan2 (*SDC2*) genes [85,86]. It is interesting to note that serum *SDC2* methylation was previously found to be highly sensitive and specific for the identification of CRC patients [119], and a recent meta-analysis including results of 12 articles confirmed the high potential diagnostic utility of methylated *SDC2* detection in stool and plasma to be used as a CRC biomarker, reporting a sensitivity and a specificity of 0.81 and 0.95, respectively [87]. Indeed, two additional tests, the iColocomf and the ColoDefense, are designed to detect methylated *SDC2* together with the *TFPI2* and *SEPT9* genes, respectively, in stool DNA [88,89]. Two tests based on the evaluation of Septin9 (*SEPT9*) in plasma, the Epi proColon^®^ 2.0 (approved by the FDA) and the ColoVantage, have also been developed [90,91]. Interestingly, methylation analysis of *SEPT9* gene in plasma DNA is also used in a test (HCCBloodTest) for the detection of hepatocellular carcinoma among patients with cirrhosis [92]. Several other peripheral biomarkers for CRC diagnosis, including methylation of *SEPT9*, *SDC2* and *VIM* [120], evaluation of *QKI* gene methylation [121], and long noncoding RNA promoter region *LINC00473* [122] in cell-free plasma DNA, have also shown high performance in early detection of affected patients, although their implementation in commercial tests has yet to be proposed. Recently, the performance of the “HelioLiver Test”, a multi-analyte blood test combining cell-free DNA methylation patterns at 77 CpG sites included in 28 genes, clinical variables, and protein tumor markers, has been tested in 247 subjects, including 122 HCC patients and 125 control subjects with chronic liver disease [93]. The test showed sensitivity and specificity for the detection of HCC of any stage of 85% and 91%, respectively [93]. Another recently proposed DNA methylation-based test for the early detection of HCC using peripheral blood samples is the “epiLiver”, which targets *CHFR, VASH2, CCNJ, GRID2IP* and *F12* genes [94].

Regarding lung cancer, an assay designed to quantify methylation of the *SHOX2* gene in bronchial aspirates and pleural effusion samples, called Epi proLung^®^, has been developed [95,96]. It should be noted that *SHOX2* analysis showed higher specificity for lung cancer when performed in bronchial aspirates than in pleural effusion samples. However, in addition to lung carcinoma, the *SHOX2* assay performed in pleural effusion samples was also able to detect cancer of other origins, including mesothelioma, gastric and intestinal cancer, lymphoma/leukemia, and breast cancer [96]. In 2017, it was developed an Epi proLung^®^ test that can be performed in peripheral blood samples (methylation of *SHOX2* and *PTGER4* genes) and that received the European in vitro diagnostic (CE-IVD) mark [97]. More recently, a test based on the methylation analysis of six genes in plasma samples (Lung EpiCheck) showed that it was able to predict lung cancer in high-risk individuals [98]. Another test, called “PulmoSeek”, which allows for the discrimination of malignant pulmonary nodules from benign ones by analyzing cell-free DNA methylation at over 100,000 CpG sites in peripheral blood samples, has recently obtained CE-IVD approval [99]. Even though several investigations have demonstrated the feasibility of DNA methylation evaluation in peripheral tissues for breast cancer diagnosis [123], commercially available tests are still lacking. However, a methylation-based CE-IVD marked assay, the Therascreen PITX2 RGQ PCR kit (Qiagen, Germany), which is designed to evaluate the methylation of the pituitary homeobox 2 (*PITX2*) gene in tumor biopsies, has been developed and commercialized. The assay is not a diagnostic one but allows to detect the breast cancer patients who could better benefit of chemotherapy based on the use of anthracyclines [124]. Two DNA methylation assays are currently available for the detection of prostate cancer. The ConfirmMDx, which is based on the evaluation of the promoter methylation levels of three genes, namely *GSTP1*, *APC* and *RASSF1* in prostate biopsy samples, was designed to identify men at lower risk for prostate cancer, thus avoiding repeated biopsies [100]. More recently, an assay that evaluates the methylation of *GSTP1, SFRP2, IGFBP3, IGFBP7, APC* and *PTGS2* genes to specifically identify individuals with a severe probability of developing prostate cancer has been proposed [101]. Moreover, a genetic and epigenetic test for bladder cancer diagnosis in patients with hematuria, which could be a sign of the presence of the tumor, has been developed [102,103]. The test, which is performed on DNA from urine and consists of the methylation analysis of *TWIST1*, *OTX1* and *ONECUT2* genes, in addition to the investigation of the mutational status of *TERT, FGFR3* and *HRAS*, could be used to substantially reduce diagnostic cystoscopies [103].

Moreover, an assay based on the evaluation of the methylation pattern of 150 loci throughout the genome has been recently developed, showing performance in detecting bladder cancer similar to that achievable by cystoscopy [104]. Recently, a CE-marked IVD test called “Bladder CARE” for the urine-based detection of bladder cancer has been developed [105]. This test is based on the evaluation of three methylation biomarkers, *TRNA-Cys, SIM2*, and *NKX1-1*, and showed a sensitivity of 93.5% and a specificity of 92.6% in detecting bladder cancer. Recently, this test also showed high sensitivity and specificity (96% and 88%, respectively) in detecting upper tract urothelial carcinoma [125]. Moreover, another test based on the methylation analysis of *ONECUT2* and *VIM* genes in urine samples for the early detection and preoperative risk stratification of bladder and urothelial cancer showed high sensitivity and specificity [106]. Two tests for the detection of cervical cancers in cervical scrapes of women with high-risk human papillomavirus (HR-HPV) infection are available in the market. They are named GynTect^®^ and QIAsure Methylation Tests and are based on the evaluation of six methylated and two methylated genes, respectively [107,108]. Furthermore, an assay designed to evaluate the *POU4F3* gene methylation in liquid-based cytology samples of HPV-positive women appeared to be a noteworthy method for cervical cancer detection and is under clinical trial evaluation [109]. In the market, there are also two CE-marked tests for the detection of cervical cancer (Cervi-M^®^) and oral cancer (Oral-M^®^) by analyzing *PAX1* and *ZNF582* gene methylation in cervix smears and oral epithelial cells [110,111]. More recently, a test called “EsoGuard”, which is based on the methylation analysis of *CCNA1* and *VIM* genes in brush cells for the detection of Barrett’s esophagus, which is the precursor and a major risk factor for esophageal adenocarcinoma, has recently received CE-IVD certification [112].

The use of high-throughput techniques that investigate DNA methylation at genome-wide levels is allowing the identification of multiple types of tumors in a single analysis, as well as the tissue of origin of cancer of unknown primary origin, i.e., cancer cases in which malignant cells are found in the body but the primary origin of the cancer is not known (Table 2).

In 2016, a diagnostic tool called EPICUP was developed to identify the tumor of origin in patients with metastatic carcinoma of unknown primary (CUP) origin [113]. The EPICUP investigation works by comparing DNA methylation data at the genome-wide level (methylome) of metastatic samples with the methylome of primary tumors for which the methylation profile is known. The authors reported that EPICUP was able to predict the tissue of origin in 87% of cases, improving the clinical outcome of the patients with this diagnosis who received a less toxic and more site- and type-directed therapy [113]. An algorithm aimed at identifying the origin of the tumor in metastatic patients has been developed by using methylome data from tumor-derived cell-free DNA in peripheral blood, named CancerLocator, to enable the diagnosis and prediction of the tissue of origin of breast, colon, kidney, liver, and lung cancer [114].

In 2020, the preliminary findings of the PanSeer assay was published, in which evaluation of blood DNA methylation was performed in subjects with a diagnosed cancer and in more than 600 asymptomatic subjects, 191 of whom received a diagnosis of tumor within four years of blood draw [115]. The authors showed that PanSeer was able to identify 5 types of cancer in 88% of confirmed cases and in 95% of asymptomatic subjects who were later diagnosed [115]. In the market, a blood-based DNA methylation test called Galleri is also available, which was designed to detect more than 50 types of cancer and able to predict the origin of cancer with high accuracy [116]. More recently, by performing sequencing of the cell-free DNA methylome in patients with colon, liver, lung, and stomach cancer, the cfMethyl-Seq assay was developed that shows high specificity and sensitivity for the detection of cancer at both early and advanced stages, as well as high performance to accurately locate the tissue from which the cancer originated [117]. In 2023, a test called OverC Multi-Cancer Detection Blood Test (MCDBT) received FDA Breakthrough Device Designation approval, a certification that expedites the development of the test and prioritizes review of subsequent regulatory submissions [118]. This test can early detect various tumors, including esophageal, liver, lung, ovarian, and pancreatic cancer, by coupling deep methylation sequencing of cell-free DNA with a machine-learning classifier of methylation patterns, enabling the detection of tumor-derived signals in plasma samples at dilution factors as low as 1 in 10,000 [118].

Overall, current data demonstrate that the implementation of DNA methylation in the oncogenic field could greatly improve patient management. The use of DNA methylation is characterizing a paradigm shift that is underway in the diagnosis of cancer, which now relies on molecular characterization rather than the traditional clinical and symptoms-based examinations. Moreover, the use of DNA methylation data will probably also change the classical approaches for patient treatments, which will no longer be based on the tumor’s anatomical origin but on the basis of the cancer molecular characterization. In this way, by using the information on methylome, mRNA, miRNA, and protein profiles of 9759 tumor samples from 33 different types of cancer from the Cancer Genome Atlas (TCGA) consortium, a “PanCancer Atlas” has been created, which allows clustering the cancers into 28 distinct molecular subtypes [126]. The PanCancer Atlas collected a huge amount of data that provides a great opportunity to better understand the pathophysiology of different types of tumors and to find common pathways as well as cancer-specific biomarkers. That information could lead to the development of effective therapies in different cancers that share a similar epigenomic profile. Furthermore, the epigenetic characterization of easy-to-obtain specimens could strongly improve the monitoring of patients under pharmacological treatment, leading clinicians to modify the therapy in those individuals who do not respond to a certain type of drug. Similarly, a deep epigenomic characterization could also lead to the discovery of new pharmaco-epigenetic biomarkers.

## 5. Conclusions and Future Perspectives

Over the period of almost one century since the discovery of DNA methylation in 1925, the scope of its study and clinical applications for the betterment of human lives has increased inch by inch. Continuous progress in our understanding of DNA methylation in various contexts of cell and disease biology has changed the course of epigenetics, with huge implications for the diagnosis of several pathological conditions such as prenatal diseases and cancers, as described in this review. Following the revolution in omics-driven biology in the last two decades, DNA methylation has come to the forefront of drug discovery, diagnosis, and basic cell and molecular biology research. However, the central dogma of molecular biology is no longer limited to genes and proteins, rather with the increase in our knowledge of post-translational modifications, astronomical complexity in proteoforms [127] is taking a pivotal role in the functional stage of a protein, the workhorse of a cell. In this crossroads of molecular biology, new mechanisms able to explain in which way DNA methylation may be undetected at the RNA level but can nonetheless be functional at the protein level have to be elucidated in depth. New concepts such as MethylMix-PA have started surfacing in the literature that can identify new genes with significant methylation effects only at the protein level and that can reveal the effect of DNA methylation on the cancer proteome [128]. Altogether, it can be said that the DNA methylation study in the course of molecular biology is entering a new era with better prospects and precise clinical applications in complex human diseases such as prenatal disorders and cancers.

## Figures and Tables

**Table 1:** DNA methylation biomarkers for cancer detection.

Type of Cancer	Sample	Technique	Epigenetic Alteration	Test Name	Sensitivity/Specificity ^1^	Phase	Reference
Colorectal cancer	Stool	MSP	*VIM* methylation	ColoSureTM	72–77% Se 83–94% Sp	CLIA	[83]
Colorectal cancer	Stool	qMSP	*BMP3* and *NDRG4* methylation	ColoGuard (Exact Sciences Co.)	92.3% Se 86.6% Sp	FDA approved	[84]
Colorectal cancer	Stool	qMSP	*SDC2* methylation	EarlyTect-Colon Cancer (Genomictree, Inc.)	90.2% Se 90% Sp	Korean Food and Drug Administration	[85]
Colorectal cancer	Stool	qMSP	*SDC2* methylation	Colosafe (Creative Biosciences)	83.8–87% Se 98% Sp	China National Medical Products Administration	[86]
Colorectal cancer	Stool	Multiplex PCR	*SDC2* and *TFPI2* methylation	iColocomf (Wuhan Ammunition Life-tech Co.)	95.31% Se 90.3% Sp	Trademark registered	[87,88]
Colorectal cancer	Stool and plasma	qMSP	*SDC2* and *SEPT9* methylation	ColoDefense (VersaBio Technologies, Inc.)	90.8% Se in stool; 45–88% Se in plasma 92.9% Sp	Trademark registered	[89]
Colorectal cancer	Plasma	qMSP	*SEPT9* methylation	Epi proColon (Epigenomics, Inc.)	75–81% Se 96–99% Sp	FDA approved	[90]
Colorectal cancer	Plasma	qMSP	*SEPT9* methylation	ColoVantage	90% Se 88% Sp	CLIA	[91]
Hepatocellular carcinoma	Plasma	rtPCR	*SEPT9*	HCCBloodTest (Epigenomics, Inc.)	85–94% Se 84–91% Sp	CE marked	[92]
Hepatocellular carcinoma	Blood	Targeted capture assay	77 CpG sites in 28 genes	HelioLiver	85% Sp 91% Se	CLIA	[93]
Hepatocellular carcinoma	Plasma	Bisulfite next-generation sequencing	*CHFR, VASH2, CCNJ, GRID2IP* and *F12* genes	epiLiver	95% Sp 84.5 Se	In development	[94]
Lung cancer	Bronchial aspirates and pleural effusion samples	qMSP	*SHOX2* methylation	Epi proLung BL Reflex Assay (Epigenomics, Inc.)	78–96% Se/96% Sp in bronchial aspirates 39.5% Se/96.2% Sp in pleural effusion	CE marked	[95,96]
Lung cancer	Plasma	MethyLight-based assay	*SHOX2* and *PTGER4*	Epi proLung^®^ blood-based version (Epigenomics, Inc.)	67–90% Se 73–90% Sp	CE marked	[97]
Lung cancer	Plasma	MSRE-qPCR	Methylation of six genes (names of the gene not available)	Lung EpiCheck (Nucleix)	56.7–87.2% Se 64.2–100% Sp	In development	[98]
Lung cancer	Plasma	Bisulfite next-generation sequencing	Over 100,000 CpG sites	PulmoSeek	41% Sp 96% Se	CE marked	[99]
Prostate cancer	Primary tissue biopsy samples	MSP	*GSTP1*, *APC* and *RASSF1* methylation	ConfirmMDX (mdxhealth)	NA	CLIA	[100]
Prostate cancer	Urine	qMSP	*GSTP1*, *SFRP2*, *IGFBP3*, *IGFBP7*, *APC*, and *PTGS2*	epiCaPture	73% Se 76% Sp	In development	[101]
Bladder cancer	Urine	MSP	*TWIST1*, *ONECUT2* and *OTX1* methylation	AssureMDX (MDxHealth)	93% Se 86% Sp	Laboratory-developed test	[102,103]
Bladder cancer	Urine	Bisulfite next-generation sequencing	150 CpG loci biomarker panel	UroMark	98% Se 97% Sp	Laboratory-developed test	[104]
Bladder cancer	Urine	qMSP	*TRNA-Cys, SIM2*, and *NKX1-1*	Bladder CARE	96.2% Sp 93.5% Se	CLIA	[105]
Bladder and urothelial cancers	Urine	Multiplex qPCR	*ONECUT2* and *VIM*	UriFind Bladder Cancer Detection Kit	85.7–89.7% Sp 88.1–91.2% Se	CE marked	[106]
Cervical cancer	Cervical scrapes	MSP	*ASTN1*, *DLX1*, *ITGA4*, *RXFP3*, *SOX17*, and *ZNF671*	GynTect^®^	65% Se 95% Sp	CE marked	[107]
Cervical cancer	Cervical scrapes	Multiplex-MSP	*FAM19A4* and miR124-2	QIAsure Methylation Test (Qiagen)	67–100% Se 68% Sp	CE marked	[108]
Cervical cancer	Liquid-based cytology	q-MSP	*POU4F3*	CONFIDENCE™ (Neumann Diagnostics)	1.67–1.74 Relative sensitivity 0.98–1.01 Relative Specificity	CE marked	[109]
Cervical cancer	Cervical scrapes	MSP	*PAX1*	Cervi-M^®^ (ISTAT BIOMEDICAL Co.)	73% Se 80% Sp	CE-marked	[110]
Oral cancer	Oral epithelial cells	MSP	*ZNF582* and *PAX1*	Oral-M^®^ (ISTAT BIOMEDICAL Co.)	72–85% Se 86% Sp	CE-marked	[111]
Esophageal cancer	Esophageal brushing	Bisulfite next-generation sequencing	*CCNA1* and *VIM*	EsoGuard	91% Sp 93% Se	CLIA	[112]

Abbreviations: CLIA, Clinical Laboratory Improvement Amendments; MSP (Methylation Specific PCR); MSRE (Methylation-Sensitive Restriction Enzyme); NA (Not Available); qMSP (quantitative MSP); qPCR (quantitative PCR). ^1^ Sensitivity and specificity values were provided by manufacturer instructions or published article.

**Table 2:** DNA methylation biomarkers for cancer of unknown origin detection.

Type of Cancer	Sample	Technique	Epigenetic Alteration	Test Name	Sensitivity/Specificity ^1^	Phase	Reference
Carcinoma of unknown primary	Tumor biopsy samples	Illumina 450/EPIC methylation arrays	Genome-wide methylation	EPICUP (Ferrer and IDIBELL)	97.7% Se 99.6% Sp	CE-marked	[113]
Multiple types of tumors	Plasma	Algorithm established from Illumina 450 methylation arrays	Genome-wide methylation	CancerLOCATOR	NA	Laboratory-developed test	[114]
Multiple types of tumors	Plasma	Bisulfite next-generation sequencing	477 genomic regions associated to 657 genes and covering 10,613 CpG sites	PanSeer (SINGLERA Genomics)	75–96% Se 96%	Laboratory-developed test	[115]
Multiple types of tumors	Plasma	Whole genome bisulfite sequencing	Genome-wide methylation	Galleri (Grail)	51.5–90.1% Se 99.00% Sp	Preclinical	[116]
Multiple types of tumors	Plasma	Bisulfite next-generation sequencing	CpG-rich cfDNA fragments	cfMethyl-Seq (EarlyDx)	74.5–80.7% Se 97.7% Sp	Laboratory-developed test	[117]
Multiple types of tumors	Plasma	Bisulfite next-generation sequencing	Genome-wide methylation	OverC Multi-Cancer Detection Blood Test (MCDBT)	52–81% Se 95–96% Sp	CE-marked	[118]

Abbreviations: NA (Not Available). ^1^ Sensitivity and specificity values were provided by manufacturer instructions or published article.

## Data Availability

Not applicable.
